# Intersecting Pathways: The Impact of Philadelphia-Negative Chronic Myeloproliferative Neoplasms on the Pathogenesis and Progression of Heart Failure with Preserved Ejection Fraction

**DOI:** 10.3390/diagnostics15162042

**Published:** 2025-08-14

**Authors:** Marius-Dragoș Mihăilă, Bogdan Caloian, Florina Iulia Frîngu, Samuel Bogdan Todor, Minodora Teodoru, Romeo Gabriel Mihăilă, Dana Pop

**Affiliations:** 1 4th Department of Internal Medicine, Department of Cardiology Rehabilitation, “Iuliu Hațieganu” University of Medicine and Pharmacy, 400012 Cluj-Napoca, Romania; dragosmihaila29@gmail.com (M.-D.M.); bogdan912@elearn.umfcluj.ro (B.C.); florina.fringu@elearn.umfcluj.ro (F.I.F.); pop67dana@gmail.com (D.P.); 2 Department of Cardiology Rehabilitation, Clinical Rehabilitation Hospital, 400437 Cluj-Napoca, Romania; 3 Department of Haematology, Faculty of Medicine, “Lucian Blaga” University of Sibiu, 550169 Sibiu, Romania; romeomihaila@yahoo.com; 4 Department of Cardiology, Faculty of Medicine, “Lucian Blaga” University of Sibiu, 550169 Sibiu, Romania; minodora.teodoru@ulbsibiu.ro

**Keywords:** heart failure with preserved ejection fraction, chronic Philadelphia-negative myeloproliferative neoplasms, echocardiography, diastolic dysfunction, *JAK2* gene mutation

## Abstract

**Background**: Heart failure with preserved ejection fraction (HFpEF) is increasingly prevalent worldwide due to ageing and comorbidities. Emerging evidence suggests that Philadelphia-negative chronic myeloproliferative neoplasms (MPNs), particularly those with *JAK2* mutations, may contribute to the development of HFpEF, especially by promoting inflammation and increasing thrombotic risk. **Methods**: This prospective case–control study assessed 58 patients with Philadelphia-negative MPNs and 41 controls, by clinical, paraclinical, and echocardiographic evaluation, to diagnose diastolic dysfunction and HFpEF according to the ESC guideline criteria. **Results**: Patients with MPNs had a significantly higher prevalence of HFpEF compared to controls (*p* = 0.008), higher H_2_FPEF scores (median 5 vs. 3, *p* < 0.001), and significant echocardiographic abnormalities, including a higher left ventricular mass index (LVMI) (100.1 vs. 76.6 g/m^2^, *p* < 0.001), E/e’ (11.00 vs. 7.00, *p* < 0.001), and pulmonary artery systolic pressure (PASP) (26.0 vs. 7.42 mmHg, *p* < 0.001). Multivariable logistic regression models identified male sex (OR = 8.993, *p* = 0.001) and the presence of *JAK2* mutation (OR = 5.021, *p* = 0.002) as independent risk factors for HFpEF in this population. **Conclusions**: Patients with chronic MPNs, particularly males and those with *JAK2* mutations, are at an increased risk of HFpEF, highlighting the importance of routine cardiologic assessment to improve outcomes in this patient population.

## 1. Introduction

With over 64 million individuals diagnosed with heart failure (HF) worldwide, it has become a significant global public health issue [1]. Recently, the lifetime risk of developing heart failure has been estimated at approximately 24% [2], indicating that one in four individuals will experience HF during their lives. Half of these cases are heart failure with preserved ejection fraction (HFpEF), which has been rising in prevalence in recent years [3]. This upward trend in the prevalence of HFpEF can be attributed to the rise in established risk factors for this type of HF [4], such as obesity, diabetes, atrial fibrillation, and demographic changes associated with increasing life expectancy.

Considering the significance of comorbidities in the progression of HFpEF, the latest European Society of Cardiology (ESC) HF management guidelines [5] recommend the active screening and treatment of comorbidities as a cornerstone of therapy for patients with this type of heart failure, alongside treatment with a sodium-glucose cotransporter 2 (SGLT2) inhibitor and management of the congestive syndrome.

While the link between cancer and HF is well established, both in relation to the neoplastic process itself and considering the cardiocirculatory and metabolic effects of chemotherapy and radiotherapy, most studies in cardio-oncology focus on the impairment of systolic function and, by extension, the occurrence of heart failure with reduced ejection fraction (HFrEF) [6]. Consequently, the connection between HFpEF and neoplastic pathology is less explored, even though the two entities share common risk factors such as obesity, hypertension, smoking, diabetes, old age, and dietary habits [7].

Moreover, most research in cardio-oncology focuses on solid tumours, leaving the role of haematological neoplastic pathology in the development of HF uncertain.

Among these, Philadelphia-negative chronic myeloproliferative neoplasms (MPNs) show an increasing prevalence [8], attributed to advances in genetic diagnostic methods and novel therapies such as protein kinase inhibitors or immune therapy [9], which improve the prognosis and survival of these patients. These MPNs comprise essential thrombocythemia (ET), polycythaemia vera (PV), and primary myelofibrosis (PMF) [10]. They lack the BCR-ABL mutation, indicating the absence of the Philadelphia chromosome, and are defined by a clonal production of hematopoietic cells [10].

Recent studies have shown that these MPNs carry a significant cardiovascular risk due to chronic inflammation, the promotion of pulmonary hypertension and atherosclerotic risk [11]. This risk is further exacerbated by genetic alterations, such as the *JAK2* gene mutation, present in 97% of patients with PV and in up to 60% of those with ET and PMF [12], and which has recently been revealed to also be present at the endothelial cell level, thereby increasing the thrombotic risk [13]. Given that both MPNs and HFpEF are characterized by a chronic proinflammatory state and endothelial dysfunction, we hypothesized that these conditions may be mechanistically linked.

In light of these new findings, this study aims to evaluate the impact of Philadelphia-negative MPNs on the development and progression of HFpEF, where the identification and treatment of comorbidities represent a key factor in the management of patients with this type of HF.

## 2. Materials and Methods

This prospective observational case–control study included 99 consecutive patients admitted to the Hematology Department of the Sibiu County Clinical Emergency Hospital between April and June 2025. They were divided into two study groups: all patients (58) diagnosed with Philadelphia-negative chronic MPNs according to the current WHO guidelines, who attended routine periodic check-ups during this period and provided informed consent, and all patients (41) without neoplastic haematological diseases or other solid neoplasms or inflammatory diseases, who also attended routine evaluations during the same period and consented to participate in the study. Patients with active or a history of solid tumours, those with an inadequate echocardiographic window for the evaluation of systolic and diastolic function, and individuals already diagnosed with HFrEF were excluded from the study. Informed consent was obtained from all subjects involved in the study.

Each patient involved in the study underwent a thorough history assessment and physical examination, focusing on possible symptoms (exertional or resting dyspnoea, fatigue, decreased exercise tolerance and other symptoms of low end-organ perfusion or volume overload), signs (pulmonary crepitations, peripheral edema, abdominal distension or ascites without a primary hepatic disease, increased jugular venous pressure, hepatojugular reflux, significant weight gain due to fluid retention, protodiastolic gallop), and risk factors for heart failure with preserved ejection fraction, as well as relevant information regarding the underlying haematological disease.

Data were collected on the values of blood count parameters at diagnosis of the haematological disorder, as well as the time since diagnosis, the type of mutation identified (if present: *JAK2*, *MPL* or *CALR*), the medical treatment, and the presence or absence of a haematological response.

Transthoracic echocardiography was performed to assess systolic and diastolic function, as well as to identify any evidence of structural or functional abnormalities consistent with a raised left ventricular filling pressure (LV mass index, relative wall thickness, LA volume index, E/e’ ratio at rest, PA systolic pressure and TR velocity at rest). The ejection fraction was assessed using the Simpson biplane method. All ultrasound examinations were performed by the same examiner, using a Siemens Acuson Juniper ultrasound machine.

The clinical risk of HFpEF was calculated using the H_2_FPEF score, which allocates 2 points for a body mass index over 30 kg/m^2^, 1 point for the presence of arterial hypertension, 3 points for paroxysmal or persistent atrial fibrillation, 1 point for echocardiographic evidence of pulmonary hypertension (PASP > 35 mmHg), 1 point for the age over 60 years, and 1 point for echocardiographic evidence of increased filling pressures (E/e’ ratio > 9).

The diagnosis of HFpEF was established according to the current ESC guidelines, based on the association of the following three criteria: signs and symptoms of HF, an ejection fraction of over 50%, and echocardiographic criteria of raised left ventricular filling pressure. We considered the echocardiographic criterion fulfilled when at least two of the aforementioned echocardiographic parameters exceeded the threshold value set by the ESC guideline. In cases with borderline findings, where advanced testing (diastolic stress echocardiography or invasive haemodynamics) was unavailable, patients were diagnosed with HFpEF if clinical signs and symptoms were clearly present alongside supportive echocardiographic evidence.

The study was conducted in accordance with the guidelines of the Declaration of Helsinki and was approved by both the Ethics Committee of the “Iuliu Hațieganu” University of Medicine and Pharmacy Cluj-Napoca (approval number AVZ135, approved date: 8 July 2024) and the Sibiu County Emergency Clinical Hospital (approval number 9051, approved date: 31 March 2025).

### Statistical Analysis

Continuous variables were expressed as medians and interquartile ranges (IQR 25–75) and compared using the Mann–Whitney U test for dichotomous dependent variables and the Kruskal–Wallis test for those with more than two categories. Categorical variables were presented as numbers (percentages %) and compared using the Pearson chi-square test; in cases where the frequency was less than or equal to 5 in any category, the Fisher exact test was applied.

A multivariable logistic regression model was applied to assess the prediction of HFpEF, and a multivariable linear regression model was used for the H_2_FPEF score. Both models were constructed using the backward (conditional) method, initially incorporating all variables, which were then gradually removed based on the lack of statistical significance, until a final model with an adequate number of variables in relation to the number of cases was achieved (applying the rule of 10 events/variable: 57 cases → maximum 6 variables in the model). The models underwent internal validation through bootstrapping with 1000 samples, and for each predictor, the B coefficient, *p*-value, and 95% confidence interval, as well as Bias-corrected and accelerated (BCa), were reported. For the statistical analysis of the data, SPSS version 26 (IBM Corporation, Armonk, New York, NY, USA) was used. The statistical significance threshold was set at α = 0.05.

## 3. Results

In this study, 57 patients diagnosed with chronic MPNs were compared to a control group of 42 individuals without MPNs, examining relevant clinical, biological, and echocardiographic differences. Table 1a illustrates the general clinical and biological characteristics of the study group. The sex distribution was similar between the groups (*p* = 0.990), with no significant differences in the analysed comorbidities, including diabetes mellitus (*p* = 0.439), hypertension (*p* = 0.513), dyslipidaemia (*p* = 0.683), or overweight/obesity (*p* = 0.970). Although the age of patients in the MPNs group was higher, it did not reach statistical significance.

Biological analyses revealed significant changes in the MPN group, consistent with the haematological profile specific to these conditions. Compared to the controls, patients with chronic MPNs exhibited notably increased values of haemoglobin (15.1 vs. 13.4 g/dL, *p* = 0.001), haematocrit (46.6% vs. 39.8%, *p* < 0.001), leukocytes (9.76 vs. 7.76 × 10^3^/µL, *p* = 0.009), neutrophils (5.98 vs. 4.91 × 10^3^/µL, *p* = 0.002), and platelets (540 vs. 275 × 10^3^/µL, *p* < 0.001).

A 1:1 individual matching (Table 1b) was performed between patients with MPN and non-MPN controls based on age (±3 years), sex, and cardiovascular comorbidities, resulting in 42 matched pairs (*n* = 84). The 17 unmatched MPN cases were subsequently included in the analysis to preserve statistical power. In the combined dataset, logistic regression (conditional method) was used, incorporating all matching variables and additional covariates to adjust for any residual confounding.

Table 2 summarises the echocardiographic and HFpEF-related characteristics of the study group. Patients with chronic MPNs showed significantly more frequent clinical manifestations of HF (*p* < 0.001), including signs and symptoms, and met the HFpEF diagnostic criteria considerably more often (*p* = 0.008). Diastolic dysfunction was markedly different between the groups, with a significantly higher proportion of MPN patients in grades 2 and 3, whereas the majority of non-MPNs patients exhibited no diastolic dysfunction (*p* < 0.001).

The H_2_FPEF score was significantly higher in the MPN group (*p* < 0.001), indicating a higher likelihood of HFpEF. Echocardiographic parameters revealed notable differences in the interventricular septal (*p* < 0.001) and posterior wall thickness (*p* < 0.001), left atrial size (*p* < 0.001), and diastolic filling parameters, including medium e’ (*p* < 0.001), E/A ratio (*p* = 0.005), and E/e’ ratio (*p* < 0.001), all suggesting more severe diastolic dysfunction and increased filling pressures in the MPN group.

Also, the LVMI (*p* < 0.001), RWT (*p* = 0.002), and PASP values (*p* < 0.001) were significantly elevated in patients with MPNs, along with a higher frequency of positive echocardiographic criteria for left ventricular diastolic dysfunction and raised left ventricular filling pressures. The total number of fulfilled echocardiographic criteria for HFpEF (Table 3) was significantly higher in the MPN group (*p* < 0.001).

The comparison of patients with chronic MPNs according to the presence or absence of HFpEF did not reveal significant differences in terms of clinical or therapeutic characteristics (Table 4). The sex distribution (*p* = 0.118) and median age (*p* = 0.538) were similar between the two subgroups. At the same time, the proportions of patients with PV, ET, PMF or unclassified forms of chronic MPNs were comparable, without relevant statistical differences.

The duration of the disease was slightly longer in patients with HFpEF, although this difference was not statistically significant (*p* = 0.487). The frequency of *JAK2* mutation and the use of various treatments (hydroxycarbamide, pegylated interferon, anagrelide, ruxolitinib) were similar in both groups. The response rate to treatment was higher in the group with HFpEF, but the difference did not reach the threshold of statistical significance (*p* = 0.144).

The analysis of clinical and echocardiographic characteristics according to the subtype of MPN (PV, ET, PMF, unclassified MPNs) revealed several statistically significant differences between groups (Table 5). The H_2_FPEF score was highest in patients with unclassified forms of MPNs, suggesting a higher clinical risk of HFpEF in this subgroup (*p* = 0.040). Additionally, left ventricular end-diastolic volumes (LVEDV) were significantly higher in patients with PMF, with the differences between groups being significant (*p* = 0.043). Systolic volumes and derived indices (SV and SI) followed the same pattern, showing higher values in PMF compared to the other subtypes (*p* = 0.023, *p* = 0.022, respectively).

**Figure 1:**
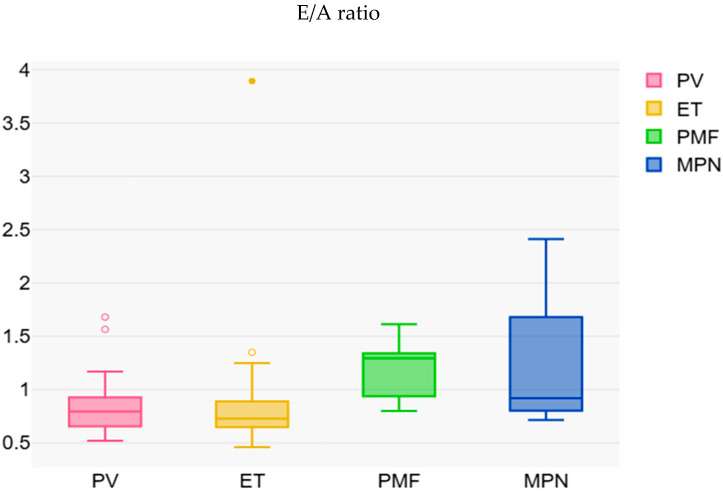
E/A ratio distribution according to the MPN type.

Other variables, such as the ejection fraction (LVEF), interventricular septal (IVS) and posterior wall (PP) thickness, as well as left atrial (LA) dimensions, varied between groups. However, most differences did not reach statistical significance, except for the A wave velocity (*p* = 0.045) and the E/A ratio (*p* = 0.025) (Figure 1), both indicating changes in diastolic filling depending on the type of MPN.

A multivariable logistic regression model was constructed for the prediction of HFpEF, adjusted for sex, age, and comorbidities. Initially, all candidate variables were included in the model, and the selection of relevant ones was conducted using the Backward Wald method (Table 6). This method involves the gradual elimination of variables that do not reach statistical significance until a stable and dimensionally adequate final form is obtained. The selection process was completed when the model incorporated six variables, respecting the rule of 10 events per variable (57/10 ≈ 6), to prevent overfitting and ensure the robustness of the estimates.

The male sex proved to be a significant predictor, with a *p*-value of 0.001 and an odds ratio (OR) of 8.993, indicating an almost nine-fold higher risk of HFpEF compared to women. The presence of the *JAK2* mutation was also significantly associated with HFpEF (*p* = 0.002), correlating with more than a five-fold higher probability of developing the condition (OR = 5.021). The other variables included in the model, namely body surface area (BSA), stroke, venous thromboembolism (VTE), and dyslipidaemia, did not reach statistical significance but were retained in the model as potentially clinically or biologically relevant factors.

To assess the robustness of the logistic regression estimates, a bootstrap procedure involving 1000 resamplings was applied (Table 7), which allows for a more precise estimation of standard errors and confidence intervals, particularly in the case of a relatively small sample. In addition to the initial B coefficients, the bootstrap bias values, adjusted standard errors, and BCa (bias-corrected and accelerated) confidence intervals were calculated.

The B coefficient for the male sex remained significant (*p* = 0.001), with a bias of 0.218 and a standard error of 0.766. The BCa confidence interval ranged from 0.570 to 4.714, which supports the robustness of the association. The *JAK2* mutation also retained statistical significance (*p* = 0.001), showing a modest bias of 0.166 and a BCa ranging from 0.380 to 3.805. The other variables—BSA, AVC, VTE, and dyslipidaemia—did not reach statistical significance in the bootstrap analysis, and their confidence intervals included the value 0, indicating uncertainty about their impact in the study population. Notably, there is a large bias and high standard error for stroke (−2.382 and 6.861), suggesting significant instability in the estimate for this variable.

In the case of the significant variables in the model–male sex and the presence of *JAK2* mutation–the bias estimated by the bootstrap method was minimal, indicating good stability of the coefficients. For the male sex, the bias was 0.218 (21.8%), indicating a slight overestimation of the B coefficient in relation to the mean of the bootstrap distribution; however, the difference is minor and does not impact the significance of the results. For the *JAK2* mutation, the bias was 0.166 (16.6%), which is also small, confirming that the coefficient estimate remains robust and consistent after resampling.

To build the multivariable model of the H_2_FPFE score, multiple linear regression was used, with the initial inclusion of all clinical variables deemed relevant. The backward selection method was utilised, involving the gradual elimination of predictors based on their statistical significance. This process continued until only those variables that significantly or relevantly contributed to the variation in the score remained in the model. In the final model, female sex was negatively and significantly associated with the H_2_FPFE score (B = −1.400, *p* = 0.001), suggesting that women had lower scores, regardless of other factors. Additionally, membership in the case group (MPNs) was positively and significantly associated with the score (B = 0.975, *p* = 0.003), indicating higher scores among patients in this group (Table 8). Leucocytosis, diabetes mellitus, history of stroke, and dyslipidaemia did not show a statistically significant association but were retained in the model due to their clinical relevance and cumulative contribution to explaining the variation in the score.

The model was internally validated using a bootstrap procedure with 1000 samples, which allowed for the estimation of the robustness of the coefficients and their variations. The bias values for each coefficient were very small (close to zero), indicating that the estimates obtained from the original model are stable and do not suffer from overcalibration or significant distortions due to resampling. For example, for the MPN variable, the bias was only 0.0000769, and for the female sex, the bias was 0.015 (practically insignificant values), which supports the internal validity of the model. These results suggest that the model is not significantly influenced by sample variations and that the coefficient estimates are reliable within the context of the studied population.

## 4. Discussion

To the best of our knowledge, the potential link between Philadelphia-negative MPNs and HFpEF has not been specifically explored in the existing literature. This gap served as a key motivation for our study. Considering the complex inflammatory and prothrombotic environment characteristic of MPNs, features that overlap with known pathophysiological mechanisms in HFpEF, we sought to investigate whether a significant clinical association exists. Identifying such a connection could help facilitate the early recognition and better management of cardiovascular complications within this patient group. We also hope this work prompts further mechanistic studies to uncover the underlying biological pathways involved.

In the present study, we highlighted a significant link between Philadelphia-negative chronic MPNs and HFpEF. Among the group with chronic MPNs, patients exhibited significantly more frequent signs and symptoms of HF, alongside echocardiographic evidence of diastolic dysfunction, increased left ventricular filling pressures, left ventricular hypertrophy, and pulmonary arterial hypertension, thereby fulfilling the ESC diagnostic criteria for HFpEF more frequently. Furthermore, given that the two patient groups (those with and without chronic proliferative neoplasms) were similar in terms of sex distribution, age, and common comorbidities associated with this type of HF, we can conclude that the results obtained may primarily reflect the effects of chronic MPNs on the development and progression of HFpEF. Moreover, as meeting the criteria for HFpEF in patients with chronic MPNs did not vary according to the type of haematological treatment administered, we can deduce that the effects on HF arise from a direct biological impact of the haematological neoplasia rather than from the therapies employed. In patients with both cardiovascular comorbidities and MPNs, distinguishing the predominant contributor to HFpEF is challenging. The temporal sequence of diagnoses may offer guidance, yet causality often remains uncertain. As recommended by the ESC guidelines, comprehensive management should include the identification and treatment of all modifiable risk factors, with the consideration of therapies such as statins, which provide anti-inflammatory and anti-atherogenic benefits.

A potential pathophysiological mechanism linking chronic MPNs and HFpEF may be systemic inflammation. Recent studies have shown that the activation of the JAK-STAT signalling pathway in chronic MPNs promotes the production of proinflammatory cytokines, leading to increased serum levels of IL-1, IL-6, IL-8, tumour necrosis factor alpha, and platelet-derived growth factor in patients with this type of haematological neoplasm, thereby inducing a significant proinflammatory state [14]. It has also been found that C-reactive protein, another marker of systemic inflammation, correlates with an increased *JAK2V617F* allele burden in patients with PV and ET [15]. Regarding HF, it has been demonstrated that chronic inflammation adversely affects myocardial structure and function, as the excessive production of pro-inflammatory cytokines elevates oxidative stress, thereby promoting the differentiation of fibroblasts into myofibroblasts that secrete collagen and degrade the extracellular matrix, consequently increasing myocardial stiffness [16]. Chronic inflammation, by reducing nitric oxide, adversely impacts the function of the sarcomeric protein titin, which exacerbates myocardial stiffness and contributes to diastolic dysfunction [17]. In our study, we assessed systemic inflammation using high-sensitivity C-reactive protein along with the NLR (neutrophil-to-lymphocyte ratio) and PLR (platelet-to-lymphocyte ratio). Although the serum levels of C-reactive protein did not differ significantly between the study groups, NLR and PLR were significantly higher in patients with chronic MPNs, who also more frequently met the HFpEF criteria. It has been proved that NLR and PLR are elevated in patients with HF, and moreover, that the increased NLR is a predictor of mortality in patients with HF [18].

Regarding the echocardiographic evidence of structural cardiac abnormalities in the context of diastolic dysfunction and increased left ventricular filling pressures, in our study we observed significantly higher values for the interventricular septum and posterior wall thickness, as well as the relative wall index (RWT) and left ventricular mass index (LVMI), in the group of patients with chronic MPNs compared to those without these conditions. In chronic MPNs, the clonal haematopoiesis that characterises these conditions results in an increase in blood viscosity [14], particularly in the case of PV. This elevation increases the afterload of the left ventricle and peripheral resistance, ultimately leading to left ventricular hypertrophy [19]. Additionally, experimental studies on transgenic mice positive for the *JAK2V617F* mutation [20] demonstrated that, due to the increased production of blood cells, these mice developed left ventricular hypertrophy alongside an increase in cardiomyocyte volume, as well as histologically evidenced extensive collagenous fibrosis.

Furthermore, in accordance with the more advanced degree of diastolic dysfunction in patients with chronic MPNs, we observed significantly higher values of the left atrial diameter and left atrial volume index (LAVI). A possible explanation for this phenomenon is that, in patients with chronic MPNs, abnormal haematopoiesis leading to an increase in blood viscosity causes the blood to behave like a non-Newtonian fluid [21], meaning it will no longer have a constant viscosity but will vary depending on the shear rate [22]. A recent study evaluating the non-Newtonian effects of hyperviscous blood by using atrial models derived from 4D CT scans [23] found an alteration in blood rheology in parallel with the increase in haematocrit, which favours stasis in the left atrium, its dilation, and thrombosis in the left atrial appendage, and consequently, the incidence of stroke.

In our cohort of patients, we also observed higher values of pulmonary artery systolic pressure (PASP) in individuals with chronic MPNs, which is consistent with the findings of other studies that evaluated the degree of pulmonary arterial hypertension assessed by ultrasound in this group of patients [24]. A recent study identified two main types of pulmonary hypertension in patients with chronic MPNs [25], namely that caused by chronic thromboembolism and group 5 pulmonary hypertension, which encompasses various complex and less understood mechanisms that cannot be classified into the other categories. Chronic thromboembolic pulmonary hypertension predominantly occurs in patients with PV and ET [25]. This condition is secondary to both the pro-inflammatory and hyperviscosity states associated with these disorders [26], as well as to endothelial changes caused by the presence of the *JAK2V617F* gene mutation, which promotes platelet aggregation and activation, subsequently enhancing the prothrombotic effect of these diseases [27]. On the other hand, group 5 arterial hypertension predominantly occurs in PMF or secondary myelofibrosis (which may represent an advanced stage of evolution in the case of PV and ET), possibly due to extramedullary haemopoiesis that can develop in the lungs and pulmonary arterioles, causing thickening of the vascular walls and increased pulmonary vascular resistance [25]. The development of pulmonary hypertension has also been identified as a negative prognostic factor for patients with chronic MPNs, with a recent study showing that in patients with PV, advanced pulmonary hypertension was independently associated with a three-fold lower survival [28].

In our analysis, we also identified significantly higher H2FPEF clinical risk scores for HFpEF in the group of individuals with chronic MPNs compared to the group without such neoplasms. Studies evaluating the prognostic value of this score in patients with chronic MPNs have found that a higher H_2_FPEF score is associated with HF hospitalisations as well as with chronic MPNs-specific risk factors, such as white blood count (WBC) and spleen size [29].

Regarding the medication used for treating chronic MPNs in our study, we did not observe any differences in the extent to which the criteria for HFpEF were met, regardless of the type of treatment administered, namely hydroxyurea, ropeginterferon alfa-2b, anagrelide, and ruxolitinib. Studies evaluating the cardiovascular effects of ruxolitinib, an oral JAK1/JAK2 inhibitor, displayed a generally neutral cardiovascular safety profile for this drug. However, a 5-year follow-up of patients included in the CONFORT-I trial revealed that congestive HF class III or IV was observed in 6.2% of patients treated for at least 48 months and 0 to 1.1% of patients treated for less than 48 months, suggesting a possible time-dependent effect of this drug on the development of HF [30]. With studies on anagrelide, an imidazoquinazoline derivative that inhibits the maturation of megakaryocytes in the bone marrow, reporting a HF or cardiomyopathy occurrence rate ranging from 0 to 3.5%, the American Heart Association states that anagrelide may cause exacerbations of HF through direct myocardial toxicity; however, patient characteristics and the primary disease make it difficult to identify patients who might be susceptible to anagrelide toxicity [31]. Regarding the effects of ropeginterferon alfa 2b on the development of HF, phase III clinical trials are underway to investigate the efficacy and cardiovascular safety of these drug in the treatment of patients with chronic MPNs [32]. However, ropeginterferon alfa 2b is contraindicated for HF patients with NYHA functional class ≥ 2.

Unlike patients without chronic MPNs, where it is known that HFpEF is more common among women [33], we observed in our group of patients that, for those with this type of hematological neoplasm, male sex represented an independent risk factor for the development of HFpEF and implied a nine-fold higher risk for developing this type of HF. This reversal of the role of sex in the development of HFpEF in patients with chronic MPNs could be explained by the fact that, in these patients, male sex is more frequently associated with PMF [34], which presents an increased risk of HF compared to the other types of chronic MPNs [35], as we also observed in our study. Recent studies have also shown that, in the case of chronic myeloproliferations, the male sex is associated with higher haematocrit levels [36] and, therefore, with higher blood viscosity. This, as previously discussed, is linked to more severe diastolic dysfunction and increased left ventricular filling pressures, which are markers of HFpEF. In these studies, male sex was also associated with a poor prognosis in chronic MPNs [34].

Another independent risk factor for the development of HFpEF that was associated with a 5-fold higher risk of developing this type of HF in our study was the presence of the *JAK2V617F* mutation. In patients with chronic MPNs, the presence of the *JAK2V617F* mutation has been shown to be a key factor in the development and progression of chronic inflammation, which is also crucial for the development of HFpEF: NLRP3 inflammasome genes are upregulated in MPN hematopoietic cells [37], and NGAL is elevated in PV, ET, and PMF, contributing to oxidative DNA damage through free radicals in *JAK2V617F* models [27]. TNF-α levels correlate with the *JAK2V617F* allelic burden, supporting neoplastic cell growth while suppressing normal haematopoiesis across MPNs [38]. Recent studies have also demonstrated that the *JAK2* gene mutation can be identified not only in hematopoietic cells but also in endothelial cells, where it enhances leukocyte adhesion, mutated endothelial cells exhibiting an upregulation of inflammatory and thrombogenic genes [13]. Inflammatory cytokines further drive thrombosis by increasing the expression of adhesion molecules (VCAM-1, ICAM-1), activating integrins, recruiting neutrophils, forming NETS, and releasing tissue factor (TF) [39]. Although endothelial dysfunction is recognised as a potential contributor to the pathophysiology of HFpEF, particularly in the context of the proinflammatory and prothrombotic state associated with MPNs, this aspect was not directly investigated in the present study. Given its potential relevance, we plan to conduct a study dedicated to the evaluation of endothelial function in Philadelphia-negative MPN patients, which may help clarify its role in the development and progression of HFpEF in this population.

NF-κB signalling, triggered by oxidative stress, promotes pro-inflammatory cytokines and chemokines [40], and oxidative stress is elevated in ET patients with the *JAK2V617F* mutation who have experienced thrombotic episodes [27]. Additionally, research indicates that clonal haematopoiesis of indeterminate potential (CHIP), driven by the presence of the *JAK2V617F* mutation, is an important determinant of outcomes in patients with HF [41]. As a member of the cytoplasmic tyrosine kinase family, *JAK* plays a crucial role in transmitting signals from cytokine receptors on the cell surface, such as the erythropoietin and thrombopoietin receptors [27]. When a ligand binds to these receptors, *JAK2* undergoes autophosphorylation and subsequently phosphorylates the receptor itself, generating docking sites for signalling proteins like STATs. In this context, a recent study in murine models of HFpEF demonstrated that empagliflozin, an SGLT2 inhibitor recommended for the treatment of HFpEF, reduces oxidative stress by inhibiting pro-inflammatory signalling in the STAT1 signalling pathway [42], further studies being needed to determine the extent to which it can reduce the risk of developing this type of heart failure in patients with chronic proliferative neoplasms.

### Study Limitations

The limitations of this study are primarily represented by the relatively small number of patients included, considering both the inclusion and exclusion criteria mentioned, as well as the comparatively low prevalence of Philadelphia-negative MPNs in the general population when contrasted with solid tumours.

Furthermore, due to the limited number of patients with PMF included in the study, it is challenging to draw conclusions regarding the impact on the development of heart failure with preserved ejection fraction compared to the other two chronic MPNs studied, namely PV and ET.

Another possible limitation of this study is that the NT-proBNP value was not determined in the patients included in the study, which could serve as an additional diagnostic criterion for HFpEF. Further studies are required to determine the correlation between echocardiographic findings and biological markers of HF in this patient population.

Furthermore, our laboratory does not routinely measure the *JAK2V617F* allele burden, so this data was unavailable for the current analysis. The absence of quantitative allele burden assessment could serve as a potential confounding factor, since different mutation loads might influence the clinical phenotype and the extent of cardiovascular involvement in patients with MPNs. Future studies including allele burden measurement could provide further mechanistic insights into the connection between MPNs and HFpEF.

Further studies could also determine the extent to which the effective treatment of Philadelphia-negative chronic MPNs improves diastolic dysfunction and elevated left ventricular filling pressures, thus preventing the development and progression of HFpEF.

Lastly, being a single-centre study, it is possible that the results obtained may be difficult to generalise to larger and more diverse populations.

## 5. Conclusions

Patients with Philadelphia-negative chronic MPNs are at a significantly increased risk of developing HFpEF, particularly male patients and those possessing the *JAK2* gene mutation. They have a significantly higher prevalence of diastolic dysfunction and manifestations suggestive of HFpEF, as reflected in both higher H_2_FPEF scores and echocardiographic parameters indicating impaired ventricular filling, left ventricular hypertrophy, and elevated pulmonary arterial pressure. Periodic cardiological evaluation, including detailed echocardiography and the H_2_FPEF score, should be integrated into the monitoring of these patients, considering the potential impact on cardiovascular morbidity and mortality.

The oncolo-haematological screening of HFpEF patients with clinical or paraclinical manifestations indicative of a Philadelphia-negative chronic MPN should be performed, as these represent significant comorbidities in the development and progression of this type of HF, as well as potential therapeutic targets whose effective management could positively influence the evolution of HFpEF.

## Figures and Tables

**Table 1:** (**a**) General clinical and biological characteristics of the study group ^a^. (**b**) Baseline characteristics after 1:1 matching between MPNs and non-MPNs ^b^.

(**a**)				
**Variable**	**Total**	**Non-MPNs (N = 42)**	**MPNs (N = 57)**	***p***
***Demographic data***				
** Sex**				0.990
** Male**	40 (40.4%)	17 (40.5%)	23 (40.4%)	
** Female**	59 (59.6%)	25 (59.5%)	34 (59.6%)	
**Age (yrs)**		59 (56–69)	67 (61–72)	0.059
**BSA (m^2^)**		1.795 (1.72–1.99)	1.85 (1.72–1.93)	0.823
***Comorbidities***				
**Diabetes mellitus**	15 (15.2%)	5 (11.9%)	10 (17.5%)	0.439
**Arterial hypertension**	82 (82.8%)	36 (85.7%)	46 (80.7%)	0.513
**Stroke**	9 (9.1%)	5 (11.9%)	4 (7.0%)	0.403
**VTE**	10 (10.1%)	4 (9.5%)	6 (10.5%)	0.870
**COPD**	5 (5.1%)	1 (2.4%)	4 (7.0%)	0.392
**CKD**	8 (8.1%)	2 (4.8%)	6 (10.5%)	0.461
**Dyslipidaemia**	57 (57.6%)	23 (54.8%)	34 (59.6%)	0.683
**Overweight/obesity**	53 (53.5%)	23 (54.8%)	31 (54.4%)	0.970
**Atrial fibrillation**	7 (7.1%)	2 (4.8%)	5 (8.8%)	0.695
**CCS**	19 (19.2%)	6 (14.3%)	13 (22.8%)	0.316
**No. of comorbidities**		2 (2–3)	3 (2–4)	0.426
***Biological analysis***	**Normal range**			
**Haemoglobin (g/dL)**	12–15 (F)	13.4 (12.1–14.2)	15.1 (13.2–17.6)	**0.001**
	13–16 (M)			
**Haematocrit (%)**	36–45% (F)	39.8 (36.9–43.3)	46.6 (40.2–52.4)	**<0.001**
	42–50% (M)			
**Leucocytes (10^3^/µL)**	4–10	7.76 (6.2–11.09)	9.76 (7.29–14.35)	**0.009**
**Neutrophils (10^3^/µL)**	2.0–7.5	4.91 (3.71–6.08)	5.98 (4.94–10.43)	**0.002**
**Lymphocytes (10^3^/µL)**	1.5–4.0	1.85 (1.5–2.74)	1.9 (1.6–2.23)	0.723
**Platelets (10^3^/µL)**	150–400	275 (239–322)	540 (443–927)	**<0.001**
**NLR**		2.51 (1.74–3.66)	3.54 (2.37–4.64)	**0.005**
**PLR**		136.41 (104.7–178)	328.5 (206.2–502.3)	**<0.001**
**CRP (mg/L)**	<5.0	2.3 (1.1–4.1)	2.03 (1.23–3.0)	0.362
**ESR (mm/h)**	<20	1 (1–8)	2 (1–9)	0.102
(**b**)				
**Variable**	**Non-MPNs (*n* = 42)**	**MPNs (*n* = 42)**	***p*** **-Value**	**SMD**
***Demographic features***				
**Sex**			0.768	0.07
** Male**	17 (40.5%)	19 (45.2%)		
** Female**	25 (59.5%)	23 (54.8%)		
**Age (yrs)**	67 (61–72)	69 (62–73)	0.261	0.15
**BSA (m^2^)**	1.84 (1.72–1.93)	1.85 (1.73–1.94)	0.612	0.05
***Comorbidities***				
**Diabetes mellitus**	5 (11.9%)	6 (14.3%)	0.749	0.07
**Arterial hypertension**	36 (85.7%)	34 (81.0%)	0.562	0.13
**Stroke**	5 (11.9%)	4 (9.5%)	0.726	0.07
**VTE**	4 (9.5%)	5 (11.9%)	0.728	0.07
**COPD**	1 (2.4%)	2 (4.8%)	0.558	0.12
**CKD**	2 (4.8%)	3 (7.1%)	0.646	0.09
**Dyslipidaemia**	23 (54.8%)	25 (59.5%)	0.670	0.09
**Overweight/obesity**	23 (54.8%)	22 (52.4%)	0.830	0.05
**Atrial fibrillation**	2 (4.8%)	3 (7.1%)	0.646	0.09
**CCS**	6 (14.3%)	5 (11.9%)	0.749	0.07
**No. of comorbidities**	3 (2–4)	3 (2–4)	0.841	0.04

^a^ MPNs, myeloproliferative neoplasms; BSA, body surface area; VTE, venous thromboembolism; COPD, chronic obstructive pulmonary disease; CKD, chronic kidney disease; CCS, chronic coronary syndrome; NLR, neutrophil-to-lymphocyte ratio; PLR, platelet-to-lymphocyte ratio; CRP C-reactive protein; ESR, erythrocyte sedimentation rate. ^b^ Data are expressed as n (%) or median (interquartile range). BSA, body surface area; VTE, venous thromboembolism; COPD, chronic obstructive pulmonary disease; CKD, chronic kidney disease; CCS, chronic coronary syndrome; SMD, standardised mean difference.

**Table 2:** Clinical and echocardiographic characteristics of the study group.

Variable	Non-MPNs (N = 42)	MPNs (N = 57)	*p*
***Clinical characteristics***			
**HFpEF**	11 (26.2%)	30 (52.6%)	**0.008**
**HF symptoms**	12 (28.6%)	41 (71.9%)	**<0.001**
**Diastolic dysfunction**			
**No disfunction**	33 (78.6%)	7 (12.3%)	**<0.001**
**Grade 1**	7 (16.7%)	15 (26.3%)	0.722
**Grade 2**	0 (0.0%)	33 (57.9%)	**<0.001**
**Grade 3**	2 (4.8%)	2 (3.5%)	0.996
**H_2_FPEF score**	3.00 (2.00–4.00)	5.00 (4.00–5.00)	**<0.001**
***Echocardiographic characteristics***			
**LVEF (%)**	60.2 (55.3–65.5)	60.1 (52.0–65.1)	0.543
**LVEDV (mL)**	84.3 (71.7–97.8)	90.2 (64.3–109.1)	0.837
**LVEDVi (mL/m^2^)**	46.7 (42.9–50.5)	47.2 (36.1–59.9)	0.788
**LVESV (mL)**	33.5 (28.0–38.6)	33.2 (26.6–44.1)	0.935
**LVESVi (mL/m^2^)**	18.8 (15.9–22.1)	19.1 (14.8–23.7)	0.718
**SV (mL)**	50.3 (44.0–62.5)	52.2 (37.2–66.9)	0.969
**SI (mL/m^2^)**	28.8 (22.3–32.1)	27.2 (22.9–36.9)	0.992
**IVS (mm)**	10.2 (8.7–11.3)	12.0 (10.0–13.0)	**<0.001**
**PW (mm)**	9.5 (8.3–9.9)	10.9 (9.4–12.0)	**<0.001**
**LVIDd (mm)**	44.8 (41.4–48.5)	45.7 (42.0–50.8)	0.494
**LVIDs (mm)**	26.7 (24.8–29.6)	27.9 (25.3–34.8)	0.079
**LA (mm)**	33.1 (31.4–35.6)	39.7 (36.7–42.2)	**<0.001**
**E (m/s)**	0.695 (0.570–0.810)	0.720 (0.600–0.830)	0.363
**A (m/s)**	0.680 (0.580–0.850)	0.810 (0.730–0.970)	**0.002**
**E/A ratio**	0.91 (0.82–1.29)	0.80 (0.69–1.01)	**0.005**
**Medium e’ (m/s)**	0.10 (0.08–0.11)	0.07 (0.06–0.08)	**<0.001**
**E/e’ ratio**	7.00 (6.13–7.87)	11.00 (8.80–12.11)	**<0.001**
**LAVI (mL/m^2^)**	32.9 (29.4–39.0)	35.8 (32.7–41.2)	**0.013**
**RWT**	0.419 (0.363–0.461)	0.455 (0.412–0.569)	**0.002**
**LVMI (g/m^2^)**	76.6 (66.3–89.2)	100.1 (82.8–114.4)	**<0.001**
**TRV (m/s)**	1.05 (1.00–2.00)	2.30 (1.50–2.80)	**<0.001**
**RAP (mmHg)**	3.0 (3.0–3.0)	3.0 (3.0–3.0)	0.058
**PASP (mmHg)**	7.42 (7.00–19.00)	26.0 (12.0–36.6)	**<0.001**

MPNs, myeloproliferative neoplasms; HFpEF, heart failure with preserved ejection fraction; HF, heart failure; LVEF, left ventricular ejection fraction; LVEDV, left ventricular end-diastolic volume; LVEDVi, left ventricular end-diastolic volume index; LVESV, left ventricular end-systolic volume; LVESVi, left ventricular end-systolic volume index; SV, stroke volume; SI, systolic index; IVS, interventricular septum; PW, posterior wall; LVIDd, left ventricular internal diameter in end-diastole; LVIDs, left ventricular internal diameter in end-systole; LA, left atrium; LAVI, left atrial volume index; RWT, relative wall thickness; LVMI, left ventricular mass index; TRV, tricuspid regurgitation velocity at rest; RAP, right atrial pressure; PASP, pulmonary artery systolic pressure.

**Table 3:** Echocardiographic criteria for the presence of left ventricular diastolic dysfunction/raised left ventricular filling pressures.

Criterion	Non-MPNs (N = 42)	MPNs (N = 57)	*p*
**E/e’**	6 (14.3%)	40 (70.2%)	<0.001
**LVMI**	7 (16.7%)	27 (47.4%)	0.001
**RWT**	21 (50.0%)	40 (70.2%)	0.041
**LAVI**	15 (35.7%)	31 (54.4%)	0.066
**TRV**	2 (4.8%)	19 (33.3%)	0.001
**PASP**	2 (4.8%)	17 (29.8%)	0.002
**No. of fulfilled criteria**	1.00 (1.00–2.00)	3.00 (2.00–4.00)	<0.001

MPNs, myeloproliferative neoplasms; LVMI, left ventricular mass index; RWT, relative wall thickness; LAVI, left atrial volume index; TRV, tricuspid regurgitation velocity at rest; PASP, pulmonary artery systolic pressure.

**Table 4:** Clinical characteristics of MPNs patients with and without HFpEF.

Variable	Total	Non-HFpEF (N = 27)	HFpEF (N = 30)	*p*
**Sex**				0.118
** Male**	23 (38.3%)	8 (29.6%)	15 (50.0%)	
** Female**	34 (56.7%)	19 (70.4%)	15 (50.0%)	
**Age, years**		67 (58–72)	67.5 (62–73)	0.538
**PV**	21 (36.8%)	10 (17.5%)	11 (19.3%)	0.998
**ET**	25 (43.9%)	11 (19.3%)	14 (24.6%)	0.963
**PMF**	7 (12.3%)	5 (8.8%)	2 (3.5%)	0.863
**Unclassified MPNs**	4 (7.0%)	1 (1.8%)	3 (5.3%)	0.755
**Disease age, months**		60 (19.5–96)	75 (15–146)	0.487
***JAK2* mutation**	52 (91.2%)	23 (85.2%)	29 (96.7%)	0.126
**Hydroxycarbamide**	28 (49.1%)	13 (48.1%)	15 (50.0%)	0.889
**Ropeginterferon alfa 2b**	11 (19.3%)	4 (14.8%)	7 (23.3%)	0.416
**Anagrelide**	15 (26.3%)	7 (25.9%)	8 (26.7%)	0.949
**Ruxolitinib**	11 (19.3%)	6 (22.2%)	5 (16.7%)	0.596
**Treatment responders**	43 (75.4%)	18 (66.7%)	25 (83.3%)	0.144
**Diabetes mellitus**	10 (17.5%)	6 (22.2%)	4 (13.3%)	0.378
**Arterial hypertension**	46 (80.7%)	23 (85.2%)	23 (76.7%)	0.416
**Stroke**	4 (7.0%)	3 (11.1%)	1 (3.3%)	0.251
**VTE**	6 (10.5%)	3 (11.1%)	3 (10.0%)	1.000
**COPD**	4 (7.0%)	2 (7.4%)	2 (6.7%)	0.913
**CKD**	6 (10.5%)	4 (14.8%)	2 (6.7%)	0.317
**Dyslipidaemia**	34 (59.6%)	16 (59.3%)	18 (60.0%)	0.995
**Overweight/obesity**	31 (54.4%)	18 (66.7%)	13 (43.3%)	0.077
**Atrial fibrillation**	5 (8.8%)	3 (11.1%)	2 (6.7%)	0.554
**CCS**	13 (22.8%)	8 (29.6%)	5 (16.7%)	0.224

MPNs, myeloproliferative neoplasms; HFpEF, heart failure with preserved ejection fraction; PV polycythaemia vera; ET, essential thrombocythemia; PMF, primary myelofibrosis; VTE, venous thromboembolism; COPD, chronic obstructive pulmonary disease; CKD, chronic kidney disease; CCS, chronic coronary syndrome.

**Table 5:** Clinical and echocardiographic characteristics of the study group, according to the chronic MPN type.

Variable	PV	ET	PMF	Unclassified MPN	*p*
**H_2_FPEF score**	3.0 (3.0–5.0)	4.0 (4.0–5.0)	4.0 (3.5–5.0)	6.0 (5.5–6.0)	0.040
**LVEF (%)**	58.6 (52.3–62.5)	57.4 (51–65.1)	64.7 (61.1–67.1)	62.98 (56.89–65.26)	0.453
**LVEDV (mL)**	75.5 (57.6–102.7)	86.3 (63.6–103)	112.4 (95.87–135.3)	96.12 (79.40–116.85)	0.043
**LVEDVi (mL/m^2^)**	41.8 (31.9–58.1)	45.7 (36.1–59.6)	61.4 (51.07–70.61)	51.92 (44.08–59.65)	0.065
**LVESV (mL)**	29 (24.9–44.6)	32.1 (28.5–36.9)	37.4 (33.91–55.3)	38.97 (29.62–45.89)	0.197
**LVESVi (mL/m^2^)**	15.2 (13.3–24.4)	19.18 (15.6–20.9)	19.7 (17.86–27.4)	21.15 (15.72–24.29)	0.345
**SV (mL)**	45.5 (34.2–60.2)	47.84(33.0–64.1)	69.1 (61–78.1)	58.70 (48.69–73.10)	0.023
**SI (mL/m^2^)**	24.1 (20.2–35.2)	27.1 (22.3–33.6)	36.9 (35.5–43.1)	31.58 (26.94–37.33)	0.022
**IVS (mm)**	12.1 (10.3–12.8)	11.7 (9.9–12.8)	11.3 (9.1–13.1)	12.74 (11.54–13.65)	0.661
**PW (mm)**	10.9 (9.2–11.9)	10.3 (8.7–12.4)	10.4 (9.7–11.6)	12.15 (10.95–12.70)	0.495
**LVIDd (mm)**	45.5 (41.6–47.2)	44.8 (38.6–50.8)	48.6 (45.9–51.2)	45.20 (40.25–49.40)	0.376
**LVIDs (mm)**	28.7 (25.8–31.4)	27.58 (23.3–35.8)	26.70(26.0–32.4)	31.15 (24.1–35)	0.992
**LA (mm)**	38.7 (36.4–40.3)	38.95 (36.6–42.2)	42.1 (41.0–45.5)	40.40 (40.1–44.3)	0.270
**E (m/s)**	0.6 (0.4–0.8)	0.695 (0.63–0.81)	0.79 (0.68–0.91)	0.895 (0.7–1.13)	0.189
**A (m/s)**	0.8 (0.7–0.8)	0.900 (0.78–1.05)	0.73 (0.57–0.77)	0.91 (0.655–1.09)	0.045
**E/A**	0.8 (0.6–0.9)	0.728 (0.64–0.89)	1.29 (1.011–1.32)	0.91 (0.80–1.7)	0.025
**Medium e’ (m/s)**	0.06 (0.05–0.07)	0.06 (0.05–0.08)	0.07(0.06–0.08)	0.07 (0.06–0.07)	0.556
**E/e’ ratio**	9.9 (8.3–11)	11.46 (9.2–12.25)	9.5 (8.6–11.9)	13.4 (11.5–16.1)	0.059
**LAVI (mL/m^2^)**	34.8 (32.7–40.6)	35.5 (32.3–41.4)	33.7 (32.7–39.8)	41.7 (39.1–48.5)	0.263
**RWT**	0.46 (0.41–0.57)	0.45 (0.39–0.58)	0.452 (0.40–0.48)	0.51 (0.44–0.63)	0.817
**LVMI (g/m^2^)**	100.9 (80.7–113.6)	98.4 (82.7–116.8)	100.49 (84.03–119.2)	103.4 (90.5–124.7)	0.952
**TRV (m/s)**	1.950 (1.3–2.5)	2.45 (2.0–2.8)	2.4 (1.6–2.8)	2.95 (2.8–3.1)	0.075
**RAP (mmHg)**	3.00 (3.00–3.00)	3.00 (3.00–8.00)	3.00 (3.00–3.00)	3.00 (3.00–5.50)	0.939
**PASP (mmHg)**	18.2 (9.7–30.5)	28.5(19–36.6)	26 (14.2–34.3)	38.1 (36.9–41.4)	0.096

MPNs, myeloproliferative neoplasms; LVEF, left ventricular ejection fraction; LVEDV, left ventricular end-diastolic volume; LVEDVi, left ventricular end-diastolic volume index; LVESV, left ventricular end-systolic volume; LVESVi, left ventricular end-systolic volume index; SV, stroke volume; SI, systolic index; IVS, interventricular septum; PW, posterior wall; LVIDd, left ventricular internal diameter in end-diastole; LVIDs, left ventricular internal diameter in end-systole; LA, left atrium; LAVI, left atrial volume index; RWT, relative wall thickness; LVMI, left ventricular mass index; TRV, tricuspid regurgitation velocity at rest; RAP, right atrial pressure; PASP, pulmonary artery systolic pressure.

**Table 6:** Multivariable logistic regression model adjusted for sex, age and comorbidities for the prediction of HFpEF in the study group.

Variable	B-Coefficient	*p*-Value	OR	95% CI for OR
**Male sex**	2.196	0.001	8.993	2.492–32.455
**BSA (m^2^)**	−1.875	0.245	0.153	0.006–3.624
***JAK2* + mutation**	1.614	0.002	5.021	11.839–13.703
**Stroke**	−0.980	0.316	0.375	0.055–2.549
**VTE**	0.733	0.362	2.082	0.430–10.077
**Dyslipidemia**	0.415	0.400	1.514	0.576–3.980

OR, odds ratio; CI, confidence interval; BSA, body surface area; VTE, venous thromboembolism.

**Table 7:** Bootstrapping internal validation of the multivariable logistic regression model adjusted for sex, age and comorbidities for the prediction of HFpEF in the study group.

Variable	B-Coefficient	Bias	Std. Error	*p*-Value	95% BCa CI
**Male sex**	2.196	0.218	0.766	0.001	(0.570–4.714)
**BSA (m^2^)**	−1.875	−0.136	1.488	0.147	(−4.757–0.698)
***JAK2*** **+ mutation**	1.614	0.166	0.598	0.001	(0.380–3.805)
**Stroke**	−0.980	−2.382	6.861	0.330	(−22.101–1.057)
**VTE**	0.733	0.239	2.491	0.374	(−2.364–5.767)
**Dyslipidaemia**	0.415	0.023	0.547	0.421	(−0.697–1.585)

BCa CI, bias-corrected and accelerated confidence interval; BSA, body surface area; VTE, venous thromboembolism.

**Table 8:** Multivariable linear regression model of H_2_FPEF score predictors.

Variable	B-Coefficient	Bias	Std. Error	*p*-Value	95% CI
**MPN+**	0.975	0.0000769	0.283	0.003	0.430–1.567
**Female sex**	−1.400	0.015	0.283	0.001	−1.940–−0.841
**WBC (10^3^/mL)**	0.017	0.002	0.022	0.392	−0.017–0.065
**DM**	0.458	0.016	0.395	0.238	−0.320–1.201
**Stroke**	−0.621	0.001	0.436	0.124	−1.585–0.198
**Dyslipidaemia**	0.526	−0.015	0.297	0.082	−0.124–1.067

CI, confidence interval; MPN+, myeloproliferative neoplasm group; WBC, white blood cell; DM, diabetes mellitus.

## Data Availability

The raw data supporting the conclusions of this article will be made available by the authors upon reasonable request.

## Abbreviations

The following abbreviations are used in this manuscript:

HF	heart failure
HFpEF	heart failure with preserved ejection fraction
ESC	European Society of Cardiology
SGLT2	sodium-glucose cotransporter 2
HFrEF	heart failure with reduced ejection fraction
MPNs	myeloproliferative neoplasms
ET	essential thrombocythemia
PV	polycythemia vera
PMF	primary myelofibrosis
WHO	World Health Organisation
LA	left atrium
LV	left ventricle
PA	pulmonary artery
TR	tricuspid regurgitation
PASP	pulmonary artery systolic pressure
IQR	interquartile ranges
BSA	body surface area
VTE	venous thromboembolism
COPD	chronic obstructive pulmonary disease
CKD	chronic kidney disease
CCS	chronic coronary syndrome
NLR	neutrophyl-to-lymphocyte ratio
PLR	platelet-to-lymphocyte ratio
CRP	C-reactive protein
ESR	erythrocyte sedimentation rate
LVEF	left ventricular ejection fraction
LVEDV	left ventricular end-diastolic volume
LVEDVi	left ventricular end-diastolic volume index
LVESV	left ventricular end-systolic volume
LVESVi	left ventricular end-systolic volume index
SV	stroke volume
SI	systolic index
IVS	interventricular septum
PW	posterior wall
LVIDd	left ventricular internal diameter in end-diastole
LVIDs	left ventricular internal diameter in end-systole
LAVI	left atrial volume index
RWT	relative wall thickness
LVMI	left ventricular mass index
TRV	tricuspid regurgitation velocity at rest
RAP	right atrial pressure
